# An Aquareovirus Exploits Membrane-Anchored HSP70 To Promote Viral Entry

**DOI:** 10.1128/spectrum.04055-22

**Published:** 2023-05-09

**Authors:** Guoli Hou, Qiushi Zhang, Chun Li, Geye Ding, Lingling Hu, Xiaoying Chen, Zhao Lv, Yuding Fan, Jun Zou, Tiaoyi Xiao, Yong-An Zhang, Junhua Li

**Affiliations:** a State Key Laboratory of Agricultural Microbiology, Hubei Hongshan Laboratory, College of Fisheries, Huazhong Agricultural University, Wuhan, China; b College of Fisheries, Hunan Agricultural University, Changsha, China; c Yangtze River Fisheries Research Institute, Chinese Academy of Fishery Sciences, Wuhan, China; d Key Laboratory of Exploration and Utilization of Aquatic Genetic Resources, Shanghai Ocean University, Shanghai, China; University of Mississippi Medical Center

**Keywords:** temperature dependent, viral entry, GCRV, HSP70, VP7

## Abstract

Temperature dependency of viral diseases in ectotherms has been an important scientific issue for decades, while the molecular mechanism behind this phenomenon remains largely mysterious. In this study, deploying infection with grass carp reovirus (GCRV), a double-stranded RNA aquareovirus, as a model system, we demonstrated that the cross talk between HSP70 and outer capsid protein VP7 of GCRV determines temperature-dependent viral entry. Multitranscriptomic analysis identified HSP70 as a key player in the temperature-dependent pathogenesis of GCRV infection. Further biochemical, small interfering RNA (siRNA) knockdown, pharmacological inhibition, and microscopic approaches revealed that the primary plasma membrane-anchored HSP70 interacts with VP7 to facilitate viral entry during the early phase of GCRV infection. Moreover, VP7 functions as a key coordinator protein to interact with multiple housekeeping proteins and regulate receptor gene expression, concomitantly facilitating viral entry. This work illuminates a previously unidentified immune evasion mechanism by which an aquatic virus hijacks heat shock response-related proteins to enhance viral entry, pinpointing targeted preventives and therapeutics for aquatic viral diseases.

**IMPORTANCE** The seasonality of viral diseases in ectotherms is a prevailing phenomenon in the aquatic environment, which causes huge economic losses every year worldwide and hinders sustainable development of the aquaculture industry. Nevertheless, our understanding of the molecular mechanism of how temperature determines the pathogenesis of aquatic viruses remains largely unexplored. In this study, by deploying grass carp reovirus (GCRV) infection as a model system, we demonstrated that temperature-dependent, primarily membrane-localized HSP70 interacts with major outer capsid protein VP7 of GCRV to bridge the virus-host interaction, reshape the host’s behaviors, and concomitantly facilitate viral entry. Our work unveils a central role of HSP70 in the temperature-dependent pathogenesis of aquatic viruses and provides a theoretical basis for the formulation of prevention and control strategies for aquatic viral diseases.

## INTRODUCTION

Grass carp (Ctenopharyngodon idella) is one of the most important aquaculture species worldwide, with over 6 million tons of annual production globally, the highest for any aquaculture species. Grass carp hemorrhagic disease (GCHD), caused by grass carp reovirus (GCRV), is one of the most damaging and contagious viral diseases, resulting in a greater than 80% mortality rate in yearly populations and huge economic losses to the grass carp aquaculture industry (1, 2). GCRV, a double-stranded RNA virus of the genus *Reoviridae*, is the first aquatic virus isolated and characterized in China. For decades, researchers and scientists have devoted much attention to studying the virus, from epidemiological studies to pathogen molecular biology and vaccine development (3, 4). In general, the peak of GCHD incidence follows the higher-temperature months from June to September, when the optimum water temperature for GCHD outbreaks ranges from 27°C to 30°C (1, 2). Thus, temperature is considered the key factor influencing GCRV transmission and pathogenesis. Additionally, it appeared that temperature had an impact on the occurrence of other aquareovirus diseases, including Atlantic salmon reovirus (TSRV) and Micropterus salmoides reovirus (MsReV) (5–8). Nevertheless, the precise mechanism of GCRV and other aquareoviruses that is highly dependent on temperature remains largely unknown.

Heat shock protein 70 (HSP70) represents an evolutionarily conserved group of molecular chaperones that are generally induced by environmental stresses (such as temperature, pH, wounding, and pathogen attack) to maintain protein quality control and cellular fitness (9). Viruses are obligate intracellular parasites that usually hijack host chaperones to fold their proteins and increase the effectiveness of infection. For mammalian viruses such as dengue virus, influenza A virus, herpes simplex virus, Zika virus, Ebola virus, hepatitis B virus, and hepatitis C virus, accumulating evidence has shown that HSP70 may participate in all stages of viral infection, including internalization of the virus particle into host cells, viral genome replication, gene expression, virion assembly, and release (10). In contrast, the role of HSP70 in aquatic ectotherms during virus infection is less well characterized and appears to be controversial. Recent studies demonstrated that upregulation of HSP70 in Procambarus clarkii and Litopenaeus vannamei after white spot syndrome virus (WSSV) infection is presumed to exert an antiviral role through enhancing the resistance of the host to WSSV (11, 12). While multiple reports suggest that HSP70 promotes viral infectivity during GCRV infection (13, 14), the exact molecular details remain largely unexplored.

Viral structural proteins play crucial roles in various stages of viral infection (15, 16). This is the case in the formation of protein complexes between the virus and host to prepare for viral entry into host cells (17). GCRV contains five core proteins and two outer capsid proteins (VP5 and VP7) in its particle (18). The outer capsid shell VP5 is believed to be key protein involving cell entry through an autocleavage-induced conformational rearrangement and removal of VP7 (3). Multiple research papers demonstrated that VP7 is the major neutralization antigen of GCRV and could be utilized as a preferred immunogen for GCHD vaccine design (19–22). In addition, VP7 can function as a major immunogen to trigger an immunoregulatory response; how VP7 participates in cell entry has not yet been fully investigated. Here, we report that HSP70 is highly involved in the temperature-dependent pathogenesis of GCRV infection. HSP70 localizes primarily on the cell membrane and interacts with the outer capsid protein VP7 during the early stage of GCRV infection. VP7 interacts with multiple housekeeping proteins to coordinate the interaction between virions and host receptors to facilitate viral entry. Our work delineates a pivotal role of cross talk between temperature-dependent molecular chaperones (HSP70) with the outer capsid shell (VP7) in determining GCRV’s early entry into cells.

## RESULTS

### Transcriptomic identification of HSP70 as a key player in GCRV infection.

**FIG 1:**
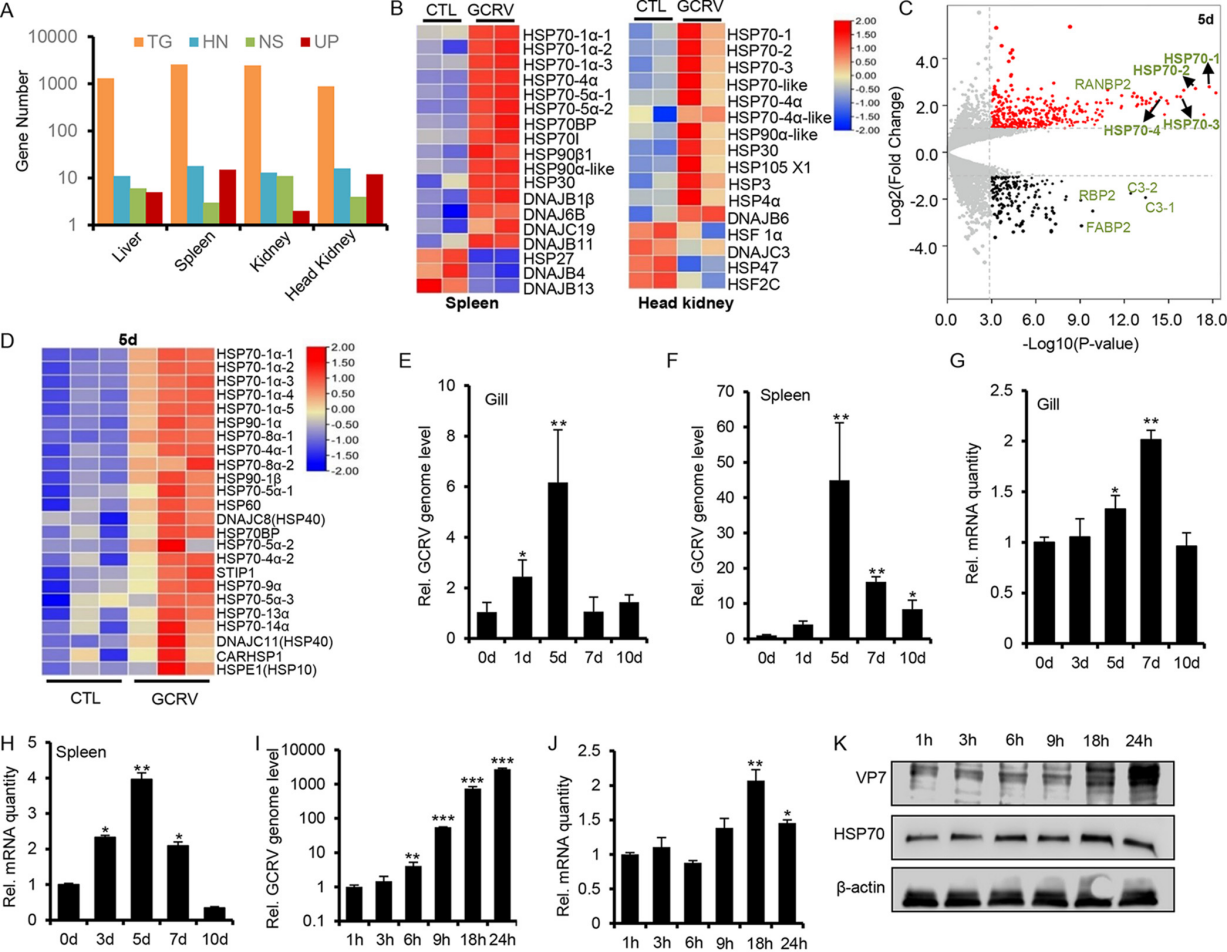
Transcriptomic identification of HSP70 as a key player in GCRV infection. (A) Numbers of genes with significant differences in transcriptomic data (NCBI accession number PRJNA759556) in different tissues from GCRV-infected grass carp. TG, total gene number; HN, HSP gene number; NG, downregulated HSP genes or nonsignificant HSP genes; UP, upregulated HSP genes. (B) Heat map analysis of transcriptomic data (NCBI accession number PRJNA759556) for HSP genes in spleen and head kidney tissues from GCRV-infected grass carp. (C) Volcano plot analysis of all genes differentially regulated from transcriptomic data (NCBI accession number PRJNA600033) in spleen tissue from grass carp infected with GCRV on the 5th day. (D) Heat map analysis of transcriptomic data for the vast majority of HSP genes from panel C. (E to H) Healthy grass carp of about 8 to 10 cm were intraperitoneally infected with GCRV. The gill and spleen tissues at 0, 1, 5, 7, and 10 days were harvested by TRIzol to analyze the replication of GCRV (E and F) and the expression of Hsp70 genes (G and H) by RT-PCR. (I to K) CIK cells were infected with GCRV (MOI ≈ 5) at 28°C and harvested by TRIzol at different time points (1, 3, 6, 9, 18, and 24 h) to analyze viral genome replication (I), HSP70 transcription (J), and the protein level of VP7 and HSP70 (K) by RT-PCR or Western blot approaches.

Viral diseases in the aquaculture industry are highly seasonal and temperature dependent, which is causing huge economic losses every year worldwide (23). In this study, we employed grass carp hemorrhagic disease (GCHD) caused by GCRV infection as a prototype to dissect the molecular mechanism behind seasonality and temperature dependency of aquatic virus infection in aquaculture. We postulate that heat shock protein (HSP) genes may be critically involved in the temperature-dependent pathogenesis of GCRV infection. The transcriptomic data (NCBI accession number PRJNA759556) from grass carp tissues (liver, spleen, kidney, and head kidney) after GCRV challenge for 5 days were analyzed to survey the differentially expressed HSP genes during GCRV infection (Fig. 1A). Among the total differentially expressed genes (1,314, 2,567, 2,468, and 890 genes in liver, spleen, kidney, and head kidney, respectively), 11, 18, 13, and 16 genes were identified as heat shock protein-related genes. In addition, 15 and 12 HSP genes in the spleen and head kidney, respectively, were significantly upregulated upon GCRV infection (*P*<0.05 [Fig. 1A]). Furthermore, among these upregulated HSP genes, HSP70 family-related genes, especially the HSP70-1α members, dominated the upregulated groups in the spleen and head kidney tissues (Fig. 1B; see also Fig. S1A in the supplemental material), suggesting that HSP70 plays a vital role in GCRV-mediated pathogenesis. To verify and refine that HSP70 (HSP70-1α in full) is involved in GCRV pathogenesis in a highly regulated manner, we reanalyzed the transcriptomic data (NCBI accession number PRJNA600033) from GCRV-infected spleen tissues from day 1 to day 7; as shown by volcano plot (Fig. 1C and Fig. S1B to D) and heat map analyses (Fig. 1D and Fig. S1E), among differentially expressed HSPs, HSP70 members were significantly upregulated, and expression peaked at day 5 postinfection (Fig. 1C and D). To further confirm that high expression of HSP70 is involved in GCRV infection, we performed real-time PCR (RT-PCR) analysis of grass carp tissues (gill and spleen) challenged with GCRV from day 1 to day 10; the data showed that the relative genome replication of GCRV in gill and spleen (Fig. 1E and F) was positively correlated with the induced expression of HSP70 (Fig. 1G and H), both of which peaked by day 5 postinfection. Similarly, RT-PCR analysis of CIK (*Ctenopharyngodon idella* kidney) cells infected with GCRV showed that the relative replication of the viral genome (Fig. 1I) correlated well with the expression of HSP70 (Fig. 1J); the viral genome replication and the expression of HSP70 both peaked at 18 h postinfection. Western blotting further confirmed that the protein level of viral capsid VP7 and HSP70 were gradually increased from 1 h to 18 h postinfection (Fig. 1K). Collectively, these data suggested that HSP70 is modulated by GCRV infection in GCRV pathogenesis.

### High expression of HSP70 and GCRV pathogenesis are temperature dependent.

**FIG 2:**
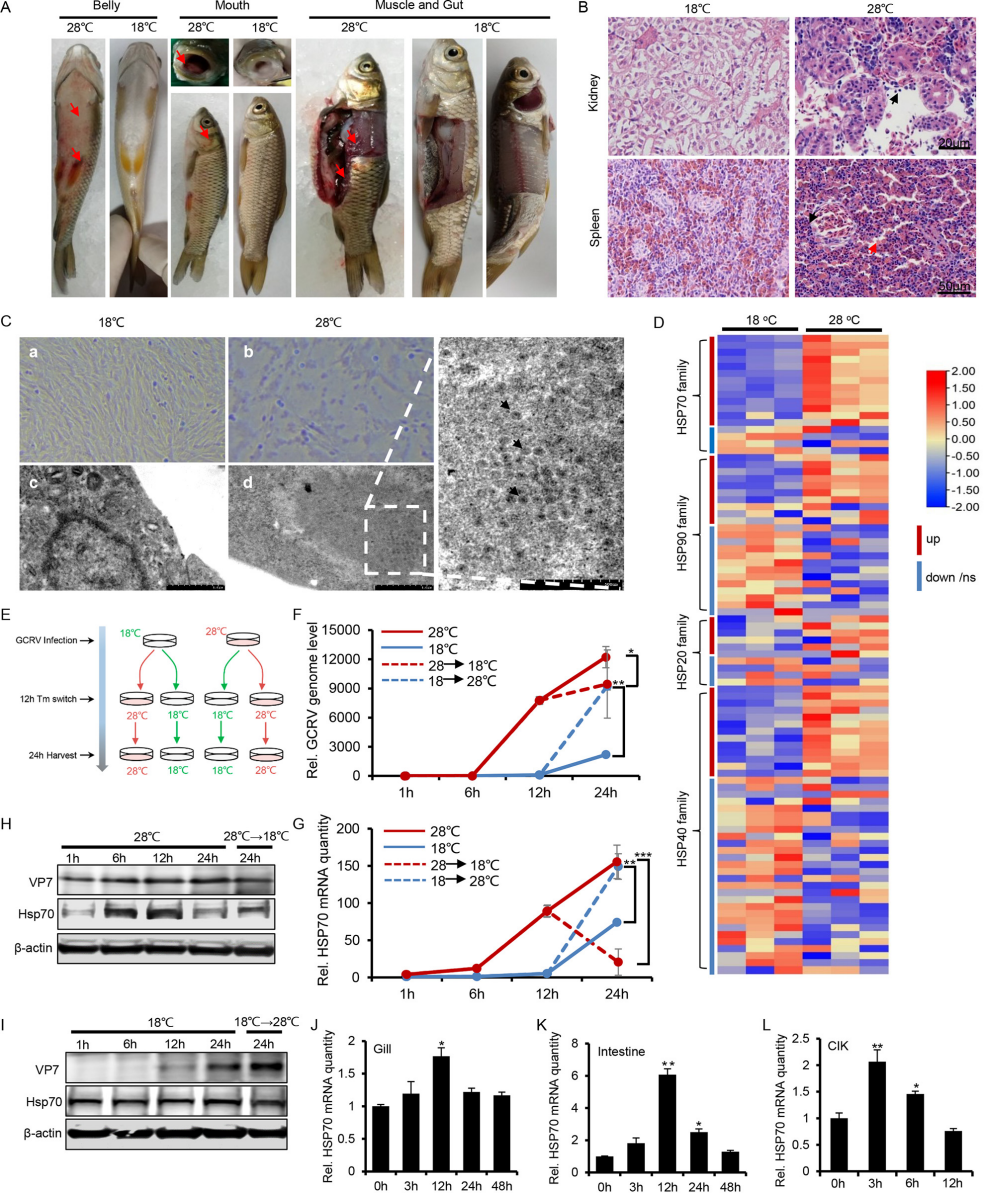
High expression of HSP70 and GCRV pathogenesis are temperature dependent. (A) Typical hemorrhagic symptoms were observed in the fins, skins, intestines, and muscles of grass carp at after GCRV infection at 28°C compared with 18°C. (B) Representative photos show histological alterations in the kidney and spleen from grass carp infected with GCRV at 18°C or 28°C and analyzed by HE staining. The black arrow and red arrow indicate the necrosis and the enlarged interspace, respectively, after infection at 28°C. (C) The cytopathic effect (CPE) and virions were observed with an optical microscope (a and b) and transmission electron microscope (c and d) in CIK cells infected with GCRV at 18°C (a and c) or 28°C (b and d). (D) CIK cells under temperature switch treatment from 18°C to 28°C were prepared for transcriptomic analysis of the HSP genes (Hsp70 family, Hsp90 family, Hsp20 family, and Hsp40 family) in the form of a heat map (NCBI accession number PRJNA862271). (E to I) Schematic of experimental design showing temperature variation affects GCRV replication and HSP70 in CIK cells (E). CIK cells were infected with GCRV at 18°C or 28°C first, followed by temperature switch treatment at 12 h from 18°C to 28°C or 28°C to 18°C, respectively, for another 12 h. After that, cell samples were harvested with TRIzol to analyze the relative GCRV genome replication (F), the transcription of HSP70 (G), and the protein level of VP7/HSP70 (H and I) by RT-PCR and Western blot approach. (J to L) Grass carp or CIK cells were prepared for temperature-switch treatment from 18°C to 28°C. The gill (J), intestine (K), and CIK cells (L) were harvested at different time points by TRIzol to analyze the transcription of HSP70 by RT-PCR.

Previous studies reported that GCRV-induced pathogenesis displayed clear evidence of temperature sensitivity and was maximal when the water temperature was 25 to 28°C, while it was greatly alleviated when the water temperature is below 20°C (24). Thus, we chose the temperatures of 28°C and 18°C to represent acute and dormant GCRV infection, respectively. As shown in Fig. 2A, the belly, fins, mouth, skin muscle, and gut from infected grass carp at 28°C showed typical symptoms of hemorrhage and inflammation, while samples from those at 18°C were clinically normal (Fig. 2A). Consistently, hematoxylin and eosin (HE) staining analysis indicated that, compared with infection at 18°C, infection with GCRV at 28°C induced apparent inflammatory pathology in the kidney and spleen of grass carp, with necrosis, enlarged interspace, and inflammatory pathological manifestations (Fig. 2B). In addition, microscopy analyses of GCRV-infected CIK cells showed that typical cytopathic effect (CPE), along with more virion particle array within cells were found during GCRV infection at 28°C, indicating that more effective viral propagation occurred with infection at 28°C than at 18°C (Fig. 2C). RT-PCR analysis further confirmed that infection at 28°C significantly increased the viral propagation in the intestine tissues and CIK cells, as well as the antiviral cytokines, compared to those at18°C (e.g., Mx and IFN3) (Fig. S2A and B). As Fig. 1 showed that HSP70 is highly involved in GCRV pathogenesis, we attempted to determine whether and how HSP70 might contribute to GCRV temperature-dependent pathogenesis. We performed a transcriptome analysis of CIK cells cultured at 18°C or transferred to 28°C from 18°C for 30 min without GCRV infection (NCBI accession number PRJNA862271). Heat map analysis revealed that 91 HSP genes from a total of 25,706 genes were differentially regulated, and the HSP70s among the HSP family were the most significantly upregulated group (Fig. 2D). The GCRV genome replication and HSP70 transcription seemed to correlate well with the infection temperature, from which the level of viral propagation and HSP70 transcription at 25°C ranked between those at 28°C and 18°C (Fig. S2C and D). To determine whether GCRV propagation and high expression of HSP70 were interrelated and temperature dependent, and endogenous HSP70 expression variation by temperature switch treatment mediates viral propagation, we first performed a temperature switch treatment, followed by GCRV infection (Fig. S2E), and the data showed that switches from 18°C to 28°C increased the HSP70 transcription and viral replication by a factor of 3 to 5, while switches from 28°C to 18°C dampened it by a factor of 7 to 9 (Fig. S2F and G). In addition, we performed GCRV infection followed by a temperature switch treatment (Fig. 2E). As shown in Fig. 2F and G, compared with infection at 18°C, GCRV infection at 28°C in CIK cells increased the viral genome replication by a factor of 7 to 20 (red solid line versus blue solid line), which correlated well with high transcription of HSP70. Western blotting further confirmed that infection at 28°C promoted the protein level of viral capsid shell VP7 and host chaperone HSP70 (Fig. 2H and I). Moreover, compared with infection at 18°C, temperature switch treatment from 18°C to 28°C boosted the viral genome replication and HSP70 transcription 20-fold and 10-fold, respectively (Fig. 2F and G, blue dashed line versus blue solid line), while 28°C to 18°C switches significantly slowed the viral genome replication and the expression of HSP70 (red dashed line versus red solid line). Consistently, Western blotting confirmed that the protein levels of VP7 and HSP70 were influenced by temperature switch treatment (Fig. 2H and I). In addition, RT-PCR analysis of CIK cells and grass carp tissues (gill and intestine) showed that temperature switch treatment from 18°C to 28°C increased the transcription of HSP70, with peak expression at 12 h (gill and intestine) or 3 h (CIK cells) after temperature switch treatment (Fig. 2J to L). Collectively, these data led us to conclude that high expression of HSP70 and GCRV pathogenesis are closely related and temperature dependent.

### HSP70 facilitates viral entry during the early stage of GCRV infection.

**FIG 3:**
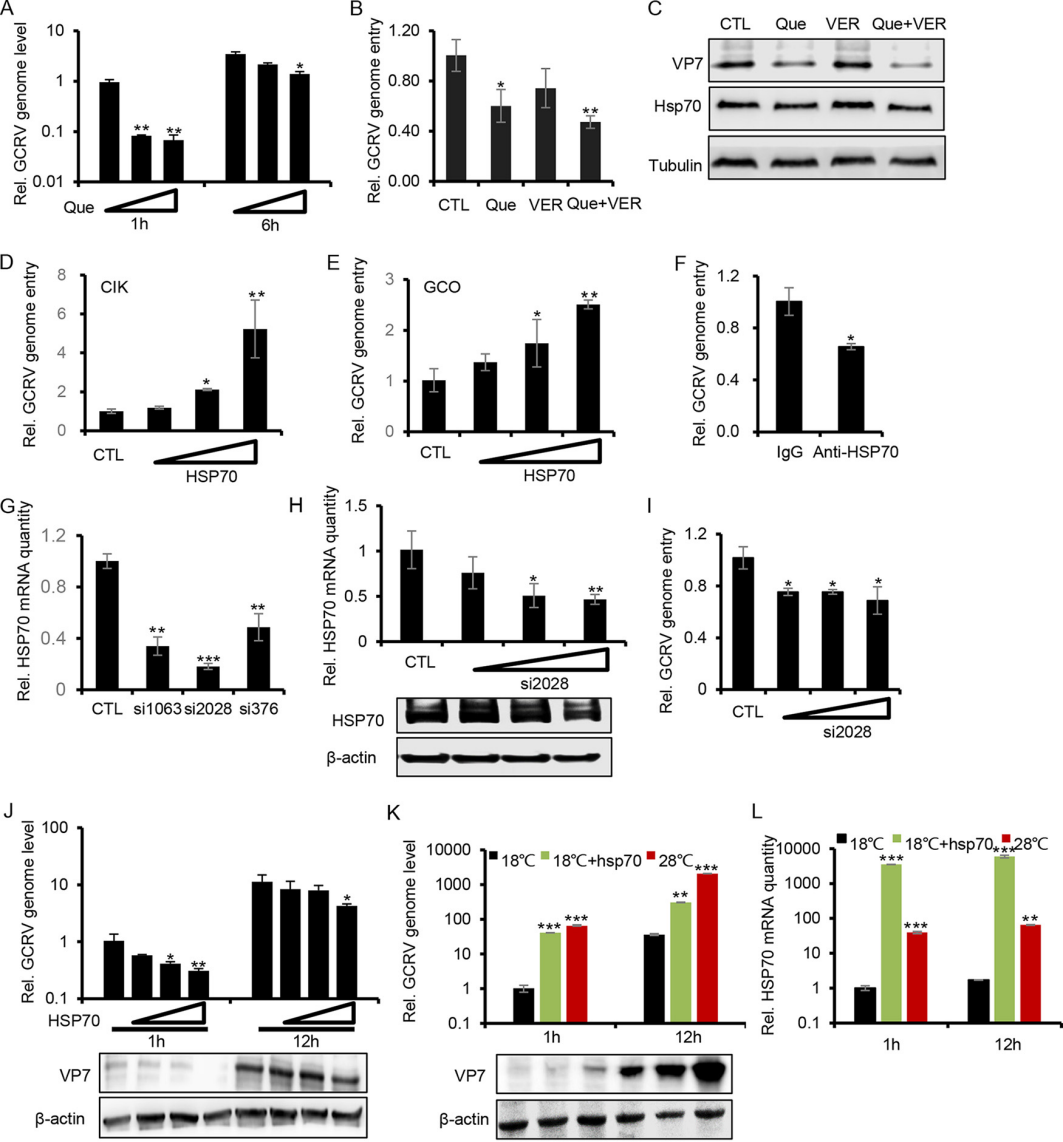
HSP70 facilitates viral entry during the early stage of GCRV infection. (A) CIK cells cultured at 28°C were pretreated with different doses of quercetin (0 μM, 1 μM, and 10 μM) for 2 h, followed by GCRV infection. Cells were then collected at 1 h and 6 h by TRIzol to examine the relative viral genome level by RT-PCR analysis. (B and C) CIK cells were preincubated with Que and/or VER for 2 h. After that, the cells were infected with GCRV for 1 h. Total RNA and protein were then separated with TRIzol to quantify the relative viral genome entry level by RT-PCR (B) and the protein level of VP7/HSP70 by Western blotting (C). (D and E) CIK cells (D) and GCO cells (E) cultured at 28°C were transfected with different doses of pEGFP-N1-HSP70 plasmid (0 μg, 0.5 μg, 1 μg, and 2 μg) for 36 h. The empty vector pEGFP-N1 was used as a control. After that, the cells were infected with GCRV for 1 h to quantify the relative GCRV genome entry level by RT-PCR. (F) CIK cells were pretreated with rabbit anti-HSP70 antibody for 2 h. After that, the cells were infected with GCRV for 1 h to quantify the relative GCRV genome entry level by RT-PCR. The control group cells were incubated with rabbit IgG. (G) CIK cells cultured at 28°C were transfected with different kinds of siRNA for 36 h. After that, the cells were collected by TRIzol to extract the total RNA and quantify HSP70 inhibition efficiency by RT-PCR. (H and I) CIK cells pretransfected with different doses of si2028 (0 μg, 0.5 μg, 1 μg, and 2 μg) were infected with GCRV for 1 h. Cells were collected by TRIzol to extract the RNA and protein. Relative expression of HSP70 (H) and viral genome entry (I) were analyzed by RT-PCR or Western blotting. (J) GCRV preincubated with different doses of HSP70 for 2 h were employed to infect CIK cells for 1 h and 12 h. Cells were then collected by TRIzol to extract the RNA and protein. Relative viral genome level was quantified by RT-PCR, while the protein expression level of VP7 was analyzed by Western blotting. (K and L) CIK cells cultured at 18°C, at 18°C with HSP70 overexpression by pEGFP-N1-HSP70 plasmid transfection, or at 28°C were infected with GCRV for 1 h and 12 h. Cells were then harvested to analyze the relative viral genome level and HSP70 transcription by RT-PCR. The protein level of VP7 was analyzed by Western blotting.

To determine the exact role of HSP70 in GCRV infection and which stage of viral infection is influenced by HSP70, we employed HSP70-specific inhibitor quercetin (Que) (25) and VER-155008 (VER) (26) to incubate CIK cells, and a cell viability assay showed that all the drugs we used under our experimental conditions had a negligible cytotoxic effect on the cells (Fig. S3A). We first utilized Que to treat CIK cells for 2 h, followed by GCRV infection for different times to examine the effect of HSP70 on GCRV infection. RT-PCR analysis showed that compared with the relative viral genome level at late stages of viral infection (6 h), the relative viral genome at the early stage of viral infection (1 h), which is assumed to be the viral genome entry level (27), was blocked to a level less than 10% by HSP70 inhibition (Fig. 3A), suggesting that HSP70 mainly participates in early viral entry step during GCRV infection (Fig. 3A). Similar results were obtained by RT-PCR assay when CIK cells were treated with Que and VER (Fig. 3B and C). Western blotting further confirmed that Que treatment reduced the entry protein level of GCRV outer capsid protein VP7, while dual treatment with Que and VER enhanced the inhibitory effect, which is likely due to reduced HSP70 synthesis by the inhibitor treatment (Fig. 3C). To avoid potential nonspecific effects caused by Que and VER, we use the HSP70 overexpression system. As shown in Fig. 3D and E, overexpression of HSP70 in CIK or GCO (grass carp ovary) cells increased the viral entry of GCRV in a dose-dependent manner (Fig. 3D and E). In addition, HSP70 blockage with antibody followed by GCRV infection further confirmed that HSP70 is required in the viral entry stage of GCRV infection (Fig. 3F). Similarly, HSP70 knockdown with small interfering RNA (siRNA) transfection, as shown by HSP70-specific siRNA screening (Fig. 3G) and knockdown efficiency verification (Fig. 3H) by RT-PCR and Western blotting (Fig. 3G to I), indicates that HSP70 is highly involved in the viral entry of GCRV infection (Fig. 3I). These results collectively showed that HSP70 facilitates viral entry during the early stage of GCRV infection.

To test whether HSP70 alone is sufficient to facilitate GCRV entry, we first used exogenous HSP70 to incubate GCRV virion particles, followed by CIK infection for 1 h and 12 h to quantify viral entry and viral genome replication, respectively. As shown in Fig. 3J by RT-PCR and Western blotting, GCRV preincubation with HSP70 significantly reduced viral genome entry (1 h) and replication (12 h) in a dose-dependent manner (Fig. 3J); in particular, the viral entry level was reduced by 70%, replicating the phenotype induced by HSP70 antibody blockage treatment, suggesting that HSP70 may function as a key coreceptor in GCRV infection. As indicated previously, temperature played an important role in HSP70 expression and GCRV pathogenesis; we examined the role of HSP70 in GCRV entry and replication at different temperatures. RT-PCR analysis showed that overexpressing HSP70 in CIK cells at 18°C, which is supposed a nonpermissive temperature for GCRV infection, significantly facilitated the viral entry, i.e., by a factor of 40, to a level comparable to that with infection at 28°C (Fig. 3K). Meanwhile, overexpressing HSP70 in CIK cells at 18°C increased the viral genome replication about 10-fold (Fig. 3K). Further Western blotting of viral structural protein VP7 validated that overexpression of HSP70 or higher temperature increased viral entry and replication (Fig. 3K). Consistently, a high level of HSP70, as shown by RT-PCR analysis in Fig. 3L, was positively related to the high level of GCRV genome entry and subsequent viral replication (Fig. 3K and L). To further verify that HSP70 upregulation by temperature stress could mediate GCRV entry, CIK cells cultured at 18°C were transiently switched to 28°C for 1 h, followed by GCRV infection, RT-PCR analysis showed that the temperature switch from 18°C to 28°C increased HSP70 transcription and GCRV genome entry by factors of 2 and 5, respectively. In contrast, temperature switch from 28°C to 18°C followed by GCRV infection reduced HSP70 transcription and GCRV genome entry by about 60% (Fig. S3B and C). Collectively, these data thus confirm our hypothesis that viral entry of GCRV is dependent on temperature and HSP70 expression.

### HSP70 localizes on the plasma membrane and interacts with the outer capsid protein VP7 of GCRV.

**FIG 4:**
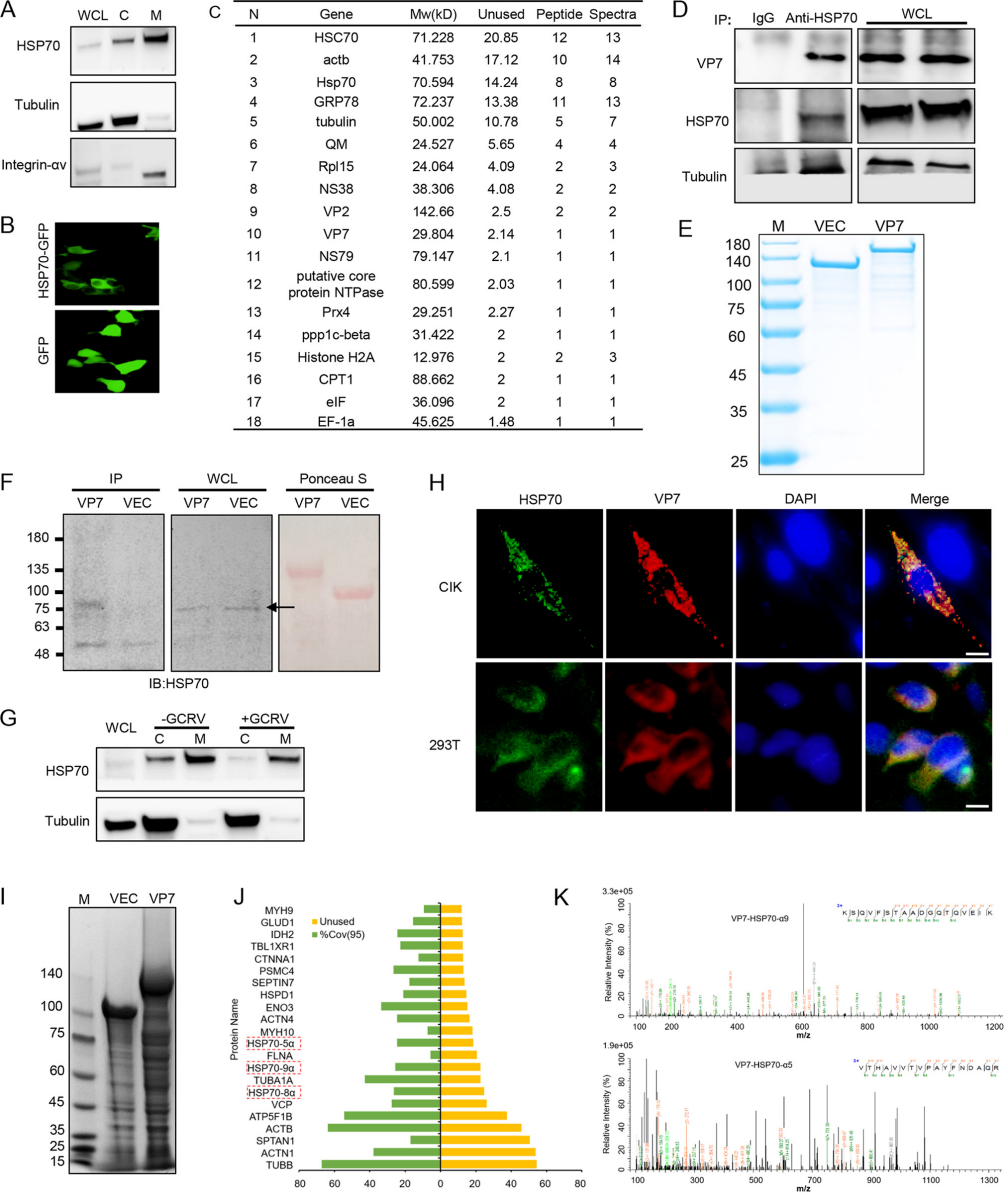
HSP70 localizes on the plasma membrane and interacts with the outer capsid protein VP7 of GCRV. (A) CIK cells cultured at 28°C were prepared to extract the cell membrane and cytoplasmic fractions with Mem-PER Plus kit (Thermo Scientific) to examine the subcellular localization of HSP70. Tubulin and integrin αv were probed as the markers of cytoplasmic and plasma membrane fractions, respectively. (B) 293T cells cultured at 37°C were transfected with pEGFP-N1-HSP70 plasmids for 36 h. Cells were then harvested to analyze the localization of HSP70 under a fluorescence microscope. pEGFP-N1 was utilized as a control. (C) CIK cells infected with GCRV were prepared for HSP70 antibody precipitation, followed by mass spectrometry analysis (project accession number PXD036008) to identify the binding candidates of HSP70. The protein list identified is ranked by the unused column, the peptide column, and the spectrogram column, which collectively reflect the protein score and the protein abundance identified in the sample. (D) CIK cells cultured at 28°C were prepared to analyze the endogenous interaction between VP7 and HSP70 by the co-IP approach. (E) Recombinant proteins VP7-HIS-EGFP-TF and HIS-EGFP-TF were purified from Escherichia coli BL21 with Ni-NAT to homogeneity and analyzed by SDS-PAGE. (F) CIK cells cultured at 28°C were prepared and lysed with IP lysis buffer. After that, cell lysates were used for incubation with VP7 beads to examine the interaction between VP7 and endogenous HSP70. (G) CIK cells cultured at 28°C with or without GCRV infection for 1 h were prepared for fractionation assay. Fractions of cytoplasmic and membrane were then incubated with His-tagged recombinant VP7. The interaction between subcellular HSP70 and VP7 was analyzed by Western blotting. (H) CIK or 293T cells overexpressing tagged VP7 and HSP70 were prepared to analyze their colocalization by immunofluorescence analysis. Scale bars, 20 μm. (I to K) CIK cells cultured at 28°C were harvested and lysed with IP lysis buffer. After this, cell lysates were incubated with VP7 beads or vector (VEC) beads to analyze the candidates of binding for VP7 from the host. Protein complexes from VP7 beads or VEC bead pulldown were separated by SDS-PAGE and stained with Coomassie blue (I). (J) Protein summary of VP7 pulldown mass spectrometry (project accession number PXD036006). The green bars indicate the unused proteins, which reflect the protein score, and the yellow bars indicate the coefficient of variation (95%), which reflects the confidence score. (K) Mass spectrometry analysis of the pulldown complex by purified VP7-HIS-EGFP identifying HSP70 α9 and HSP70 α5 as the targets of VP7.

To determine the mechanism of how HSP70 facilitates viral entry in GCRV infection, we hypothesized that HSP70 of grass carp may localize on the plasma membrane to interact with key viral structural proteins to help viral entry during the early phase of GCRV infection. Fractionation analysis of CIK cells showed that HSP70 was mainly distributed on the plasma membrane and partially on the cytoplasmic fraction (Fig. 4A). Immunofluorescence analysis further confirmed the plasma membrane localization of HSP70 when overexpressed in HEK293T (293T) cells (Fig. 4B). To identify the potential target viral proteins of HSP70 engaging viral entry during the early stage of GCRV infection, we performed a coimmunoprecipitation assay with an HSP70 antibody followed by mass spectrometry (MS) analysis (project accession number PXD036008), the data showed that transcription- and metabolism-related proteins (e.g., EF-1α, Rpl15, GRP78, CPT1, and Prx4), cytoskeleton proteins (e.g., β-actin, α-actin, α-tubulin, and myosin), and several viral proteins (e.g., NS38, VP2, VP7, and NS79) were identified as candidate target proteins of HSP70 (Fig. 4C). VP7 is one of the major outer capsid proteins of GCRV and has previously been proven to function as a key neutralizing antigen for GCRV infection and immune escape (19, 21, 22, 28). Nevertheless, the role of VP7 in the viral early phase of infection remains largely unknown. We thus hypothesized that HSP70 may interact with VP7 to facilitate viral entry into host cells during the early phase of GCRV infection. Endogenous coimmunoprecipitation analysis with HSP70 antibody showed that HSP70 and VP7 formed a complex during the early stage of GCRV infection (Fig. 4D). In addition, we purified His-tagged recombinant VP7 to homogeneity as bait (Fig. 4E), and pulldown analysis confirmed that recombinant VP7 interacted with endogenous HSP70 in CIK cells (Fig. 4F). To test whether VP7 interacts with HSP70 on the plasma membrane, we separated the fractions of cytoplasmic and plasma membrane from CIK lysates and incubated the fractions with nickel-nitrilotriacetic acid (Ni-NTA) beads coupled with recombinant VP7, His pulldown analysis confirmed that VP7 and HSP70 mainly colocalized on the plasma membrane of CIK cells, while GCRV infection increased their interaction on the membrane (Fig. 4G). In addition, immunofluorescence analysis further confirmed the colocalization between HSP70 and VP7 in CIK cells and 293T cells upon their cotransfection (Fig. 4H). Moreover, mass spectrometry (project accession number PXD036006) analysis of VP7 pulldown complex from CIK lysates further confirmed that the HSP70 family, along with multiple cytoskeletal proteins (e.g., tubulin, actin, and myosin), constituted the main targets of VP7 (Fig. 4I to K). Taken together, these data collectively led us to conclude that HSP70 primarily localizes on the plasma membrane and interacts with the outer capsid protein VP7 of GCRV.

### VP7 functions as a coordinator protein to promote GCRV entry.

**FIG 5:**
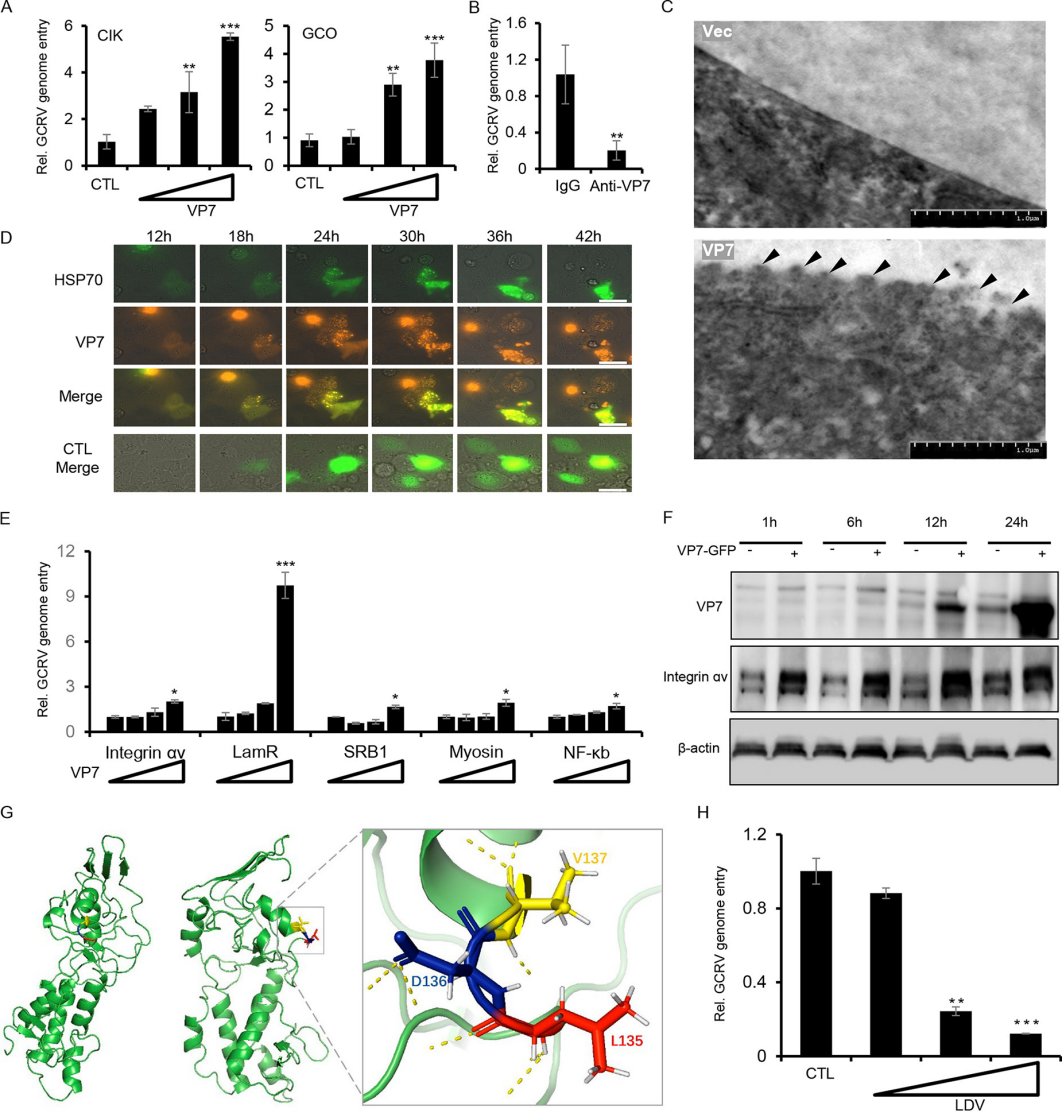
VP7 functions as a coordinator protein to promote GCRV entry. (A) CIK cells and GCO cells pretreated with different doses of recombinant VP7 were infected with GCRV for 1 h and prepared to analyze the relative viral genome entry by RT-PCR analysis. (B) GCRV particles were pretreated with mouse anti-VP7 antibody for 2 h. After that, CIK cells were infected with pretreated GCRV for 1 h. CIK cells were then harvested by TRIzol to quantify the viral genome entry by RT-PCR analysis. The control group cells were incubated with rabbit IgG. (C) CIK cells were pretreated with recombinant VP7 for 2 h to analyze the morphological and structural changes of the cells by electron microscopy analysis. The control group was pretreated with the protein expressed by an empty plasmid. (D) GCO cells overexpressing tagged VP7 and HSP70 were prepared to analyze their colocalization and state of existence of the proteins in GCO cells at different time points by fluorescence analysis. Scale bars, 20 μm. (E) CIK cells pretreated with different doses of recombinant VP7 for 2 h were infected with GCRV for 1 h and harvested to quantify the relative expression of plasma membrane-related genes by RT-PCR analysis. (F) CIK cells were incubated with purified recombinant protein VP7-EGFP for 2 h. After that, cells were washed 3 times with PBS, followed by GCRV infection for 1 h. Cells were collected to analyze the expression of VP7 protein and integrin αv by Western blotting. (G) The 3D structure of VP7 protein was predicted with Swiss model. The LDV sequence of VP7 protein was edited and shown with PyMOL. (H) CIK cells pretreated with different doses of LDV peptide were infected with GCRV for 1 h. After that, cells were collected and prepared to analyze the relative viral genome entry by RT-PCR analysis.

To examine the role of VP7 in the early phase of GCRV infection, we preincubated CIK cells or GCO cells with purified VP7 protein; GCRV genome entry quantification by RT-PCR analysis showed that VP7 promoted the viral entry in a dose-dependent manner (Fig. 5A). The proviral function of VP7 is likely dependent on its full-length sequence, while the lack of an N terminus or C terminus is unable to promote the viral infection (Fig. S4A). In addition, compared with VP7 only, coincubation of VP7 with VP5 reduced the viral entry into host cells, which is likely related to HSP70 expression (Fig. S4B). Conversely, VP7 blockage by VP7 antibody treatment inhibited the viral entry of GCRV infection to a level approximately 20% that of the control group (Fig. 5B). To further explore the underlying mechanism of VP7 in viral entry, we performed an electron microscopy analysis of CIK cells preincubated with purified VP7 protein. As shown in Fig. 5C, CIK cells treated with VP7 exhibited fuzzy and wrinkled structures on the surface of cells, while the surface of control cells was rather smooth and uniform (Fig. 5C), suggesting that VP7 may induce a morphological change of host cells through reshaping the cellular behaviors, such as assembling relevant molecules into functional complexes and cytoskeletal rearrangement, assisting the cells ready for viral entry. Consistent with this hypothesis, live-cell imaging analysis of GCO cells stably coexpressing VP7 and HSP70 exhibited clear colocalization, from which scattered aggregate signals were observed at first and fused into bigger condensates over time (Fig. 5D and Videos S1 and S2). In addition, mass spectrometry analysis of the VP7 pulldown complex from CIK lysates identified several cargo proteins and housekeeping proteins (e.g., HSPs, actin, tubulin, and myosin) as substrates (Fig. 4J). Interestingly, RT-PCR analysis showed that CIK cells incubated with VP7 appeared to activated NF-kB signaling and increase the transcription of multiple receptor-related genes, such as genes for integrin-αv, LamR, myosin, and SRB1 (Fig. 5E). Western blotting further confirmed that exogenous VP7 incubation in CIK cells significantly increases the protein level of integrin-αv expression and viral propagation (Fig. 5F), and overexpression of VP7 in CIK cells induced the transcription of HSP70 in a dose-dependent manner (Fig. S4C). Moreover, three-dimensional structure modeling of VP7 protein by SWISS-MODEL showed that VP7 assembled as a rod-like shaped morphology, and an LDV tripeptide was observed on the surface of VP7 protein structure and located at the interface between an α-helix and a linker-loop (Fig. 5G). LDV has been reported to function as the motif of virus-integrin binding (13), suggesting that VP7 may interact with genuine receptor integrins to promote viral entry. Consistently, pretreating cells with LDV tripeptide blocked the viral entry of GCRV infection in a dose-dependent manner (Fig. 5H). Altogether, these data collectively implied that VP7 may function as a key coordinator protein, bridging the virus-host interaction platform, interacting with coreceptor HSP70 and other receptors on the plasma membrane during the early phase of GCRV infection, reshaping the cellular behavioral rearrangement, and concomitantly promoting viral entry in GCRV infection.

## DISCUSSION

**FIG 6:**
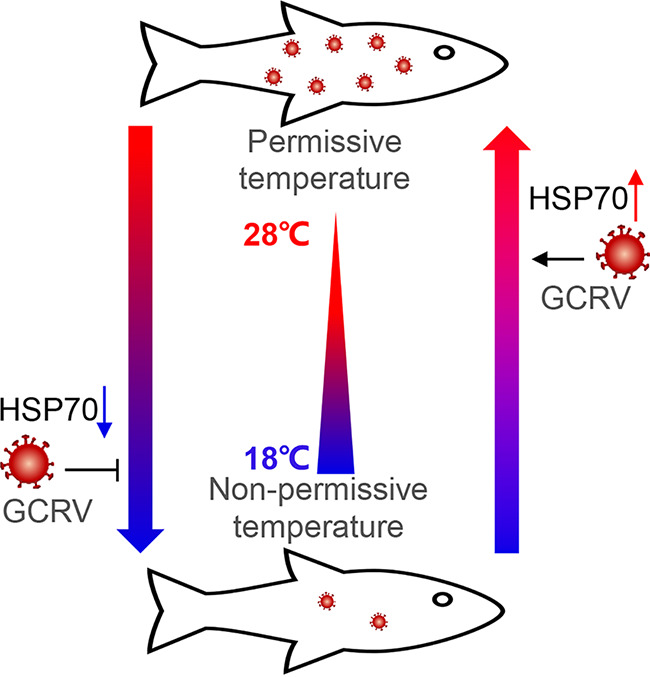
Model depicting that heat shock protein HSP70 is exploited by GCRV to mediate temperature-dependent pathogenesis of viral infection. Compared with nonpermissive temperatures (e.g., 18°C), GCRV infection at permissive temperatures (e.g., 28°C) induces the expression of primary plasma membrane-anchored HSP70 to facilitate viral entry into host cells. Temperature stress from nonpermissive temperatures to permissive temperatures increases the expression of HSP70 to benefit viral infection, while a switch from permissive temperatures to nonpermissive temperatures lowers the expression of HSP70 to dampen the viral infection.

Aquatic viral diseases are often temperature and season dependent, but our understanding of the underlying molecular mechanism of the temperature dependency of viral pathogenesis in the aquaculture industry is still in its infancy, which has impeded the development and innovation of strategies to prevent and control the diseases. Here, we report that HSP70 plays a vital role in viral entry in GCRV infection. Mechanistically, we demonstrate that the expression of HSP70 changes rapidly in response to the environmental temperature, and the primary plasma membrane-localized HSP70 is exploited by GCRV during the early phase of infection. HSP70 functions as a key chaperone molecule via tight interaction with viral outer capsid protein VP7, which forms a complex to induce and recruit other membrane receptors involved in GCRV entry (e.g., integrin, LamR, SRB1, and myosin) to facilitate viral entry into host cells (Fig. 6).

HSP70s are a family of highly conserved, ubiquitously expressed chaperone molecules that are found in all living organisms and function as survival proteins through mounting cytoprotective roles against cellular stresses, such as elevated temperature, hypoxia, microbial infection, and diseases (14). An increase in HSP70 expression following viral infection has been widely observed, and emerging evidence suggests that HSP70 systems are potentially involved in all phases of the viral life cycle, including cell entry, virion disassembly, transportation, replication and transcription of the viral genome, morphogenesis of virion particles, and cell-to-cell transmission (10, 14). Fishes are ectotherms, and viral diseases in aquaculture are prone to be influenced by environmental factors, such as temperature, ammonia nitrogen, dissolved oxygen, and pH. Although multiple reports so far have described the involvement of HSP70 in aquatic viral diseases, the role and mechanism of HSP70 during viral infection in ectotherms are still controversial (11, 12, 29–32). Our data show that HSP70 is a key factor in GCRV infection, suggesting that HSP70, among the most conserved protein in all organisms, shares functions similar to the mammalian HSP70-virus interaction (10, 14). Nevertheless, as fishes are ectotherms, our data were restricted to the experimental conditions we tested. Grass carp is a representative eurythermic fish; such fishes sense and respond to water temperature by adjusting their physiology, metabolism, and immune response (33). We cannot exclude the possibility that HSP70 in grass carp may play an antiviral role when outside the temperature range of 18°C to 28°C. Likewise, it was recently reported that during the processes known as thermotherapy and fever, elevated expression of HSPs in response to higher temperature could inhibit viral replication through immune response regulation (34–36). Future studies concerning the role of HSP70 beyond 30°C or below 18°C during GCRV infection will help us better define the complicated HSP70-virus relationship in aquaculture.

GCRV is the first aquatic virus identified and investigated in China since the 1970s, and our understanding concerning the pathogenesis of GCRV entry, especially the temperature dependency-related molecular mechanism, is far from comprehensive. Some previous studies suggested that HSP70 has a proviral effect in facilitating the entry stage of GCRV infection (13, 14), while the mechanism remains largely unknown. Deploying multiomics, molecular biology, and biochemistry analyses, we delineated the mechanism as follows: temperature-dependent and primarily plasma membrane-localized HSP70 interacts with outer capsid protein VP7 from GCRV virion particles, which recruits integrin and reshapes the host cells to facilitate viral entry. Our work provided further credence concerning the key role of HSP70 in GCRV entry. Interestingly, preincubation of CIK cells or GCRV virion particles (data not shown) with HSP70 antibody impeded the viral entry, implying that HSP70 is probably incorporated into the virion particle to increase the infection efficiency, which is a common strategy used by viruses (37–39). Future mechanistic studies of the temperature-dependent GCRV virion infectivity may provide more insight. In addition, we demonstrated that the outer capsid protein VP7 of GCRV may function as a key coordinator protein to bridge the virion and host cells. VP7 interacts directly or indirectly with multiple housekeeping proteins, such as α/β-tubulin, α/β-actin, glyceraldehyde-3-phosphate dehydrogenase (GAPDH), myosin, HSPs, RNA polymerase related proteins, ribosomal proteins, citric acid cycle-related proteins, and transportation-related proteins, which are essential and crucial for the maintenance of basic cellular function (40). Additionally, VP7 activates NF-κB signaling, associates with plasma membrane-anchored receptor integrin, and confers a morphological change on infected CIK cells. These results collectively imply that VP7 affects many fundamental cellular processes, consistent with the observation that VP7 displays good antigenicity and immunogenicity, and is an ideal candidate for the development of GCRV subunit vaccine (19–22, 28). Nevertheless, it is unclear how VP7 mechanistically reshapes the host’s behaviors to play an integrated role, which is an intriguing question and worthy of further study in the future. Taken together, our findings uncover a mechanism by which GCRV hijacks temperature-dependent HSP70 on the membrane to interact with VP7 to enhance viral entry. Our work sheds new light on the mechanistic study of temperature-dependent aquatic viral diseases and lays the foundation for developing targeted prevention strategies for grass carp hemorrhagic disease.

## MATERIALS AND METHODS

### Ethics statement.

The animals used in this study include grass carp, which are approved by the Ethics Committee of Hunan Agricultural University, and the animal experiments were carried out in accordance with animal ethics guidelines.

### Fish.

Gender-random grass carp of about 8 to 10 cm were raised temporarily in the recirculating system at 28°C or 18°C for GCRV infection or temperature switch experiments.

### Cell lines and virus.

CIK (*Ctenopharyngodon idella* kidney) cells were cultured in medium 199 (M199) containing 10% fetal bovine serum (FBS), penicillin (100 U/mL), and streptomycin (100 μg/mL) at 28°C in a carbon dioxide-free atmosphere. GCO (grass carp ovary) cells and HEK293T (human embryonic kidney cells) were maintained in Dulbecco’s modified Eagle’s medium (DMEM) supplemented with 10% FBS, penicillin (100 U/mL), and streptomycin (100 μg/mL) at 28°C and 37°C, respectively, in a humidified 5% CO_2_ atmosphere. Grass carp reovirus (GCRV) was proliferated in CIK cells. The sources of cell lines and virus are detailed in Table S1 in the supplemental material.

### Temperature stress experiment.

Grass carp of about 8 to 10 cm were raised in the recirculating system at 18°C for 1 week of adaptation. Next, the fish were transferred to a tank at 28°C. Tissues, including gill and intestine, were collected at 0 h, 3 h, 12 h, 24 h, and 48 h for RT-PCR analysis. CIK cells were cultured at 18°C or 28°C for 48 h of adaptation. After that, GCRV infection and temperature switching experiments from 28°C to 18°C or 18°C to 28°C were conducted. Cell samples were treated with TRIzol at different time points for RT-PCR analysis.

### DNA transfection.

pEGFP-N1-HSP70, pEGFP-N1, pDsRed-N1-VP7, or pDsRed-N1 plasmid transfections were carried out using Invigentech INVI DNA RNA according to the manufacturer’s protocol. CIK, GCO, or 293T cells were seeded into 6-well plates to be 70% confluent before transfection. After that, 3 μg of plasmid and 3 μL of Invigentech INVI DNA RNA were mixed and incubated for 15 min; then DNA-Invigentech INVI DNA RNA complex was gently added to cells and incubated for 24 h at 28°C. Then the medium was removed and complete DMEM was added to cells, which were cultured in incubators for another 12 to 24 h as required.

### Quantitative RT-PCR.

Total RNA from tissues and cells was extracted using RNA isolater total RNA extraction reagent (Vazyme). After that, cDNA was synthesized by the ReverAid first-strand cDNA synthesis kit (Thermo Scientific) following the manufacturer’s protocol. Real-time PCR (RT-PCR) was performed with SYBR master mix (Vazyme). The relative mRNA expression level for target genes was analyzed by the threshold cycle (2^−ΔΔ^*^CT^*) method using the β-actin gene as a reference gene. Primer sequences are listed in Table S2.

### Subcellular fractionation.

Cells were treated with the Mem-PER Plus kit (Thermo Scientific) following the manufacturer’s protocol. Briefly, CIK cells (2 × 10^6^) were collected with a cell scraper and washed 3 times with 3 mL of cell wash solution. The cell pellet was suspended with 0.75 mL of permeabilization buffer (with 1 mM phenylmethylsulfonyl fluoride [PMSF]) for incubation for 10 min at 4°C with constant mixing. Permeabilized cells were centrifuged for 15 min at 16,000 × *g* to get the supernatant containing cytosolic protein fractions. After that, 0.5 mL of solubilization buffer was added to the pellet and incubated at 4°C for 30 min with constant mixing. Membrane and membrane-associated fractions were obtained by centrifuging the solubilization solution at 16,000 × *g* for 15 min at 4°C.

### Inhibitor assay.

CIK cells were pretreated with different concentrations of quercetin (dissolved in dimethyl sulfoxide [DMSO] at 0 μM, 1 μM, or 10 μM), VER-155008 (dissolved in DMSO at 2 μM), or LDV peptide (0 μg, 4 μg, 8 μg, or 16 μg) for 2 h. After that, the cells were infected with GCRV for 1 h and 12 h to examine the relative viral genome entry and replication, respectively.

### Western blotting.

CIK cells in a six-well plate were collected and lysed with 200 μL/well of IP lysis buffer (Beyotime) containing 1 mM PMSF, followed by whole-cell lysate centrifugation at 12,000 rpm and 4°C for 20 min. Supernatant was resolved with 5× SDS buffer and boiled for 10 min at 98°C. Protein samples were separated by SDS-PAGE, followed by transfer to a polyvinylidene difluoride (PVDF) membrane. The membranes were blocked with a solution of 4% skimmed milk in Tris-buffered saline with Tween 20 (TBST) for 1 h by gentle shaking at room temperature. After that, the indicated primary antibodies (1:1,000 to 1:2,000) were incubated for 8 h at 4°C and horseradish peroxidase (HRP)-conjugated secondary antibodies (1:2,000 to 1:4,000) for 1 h at room temperature. Blotting images were visualized using an ECL detection kit (Beyotime).

### siRNA assay.

Three short siRNAs targeting gcHSP70 were designed by Sangon Biotech Company (Shanghai, China). These siRNAs included siRNA376 (sense, 5′-CCAAAGGUUAAGUCGAAUTT-3′; antisense, 5′-AUUCGACUUGAACCUUUGGTT-3′), siRNA2028 (sense, 5′-GGACUGAUAUCAAUCUCUUTT-3′; antisense, 5′-AAGAGAUUGAUAUCAGUCCTT-3′), and siRNA1053 (sense, 5′-GGACAAGUCUCAGAUCCAUTT-3′; antisense, 5′-AUGGAUCUGAGACUUGUCCTT-3′). A control siRNA (NC) that has no homology with grass carp HSP70 (gcHSP70) mRNA was used as a control. CIK cells were transfected separately with gcHSP70 siRNA or NC using Invigentech INVI DNA RNA according to the instructions of the manufacturer. At 24 h after transfection, CIK cells were infected with GCRV at 28°C for different times to analyze the viral genome entry or replication by RT-PCR.

### Coimmunoprecipitation (co-IP).

CIK cells were collected and lysed with cell lysis buffer supplemented with PMSF (1 mM) following the manufacturer’s protocol. Then whole-cell lysates were centrifuged at 12,000 rpm for 20 min at 4°C. The precipitate was discarded, and protein A/G agarose beads (20 μL) were added into the supernatant at 4°C for 1 h for precleaning. After that, the whole-cell lysates were centrifuged at 2,000 rpm for 2 min at 4°C to discard the agarose beads, and the supernatant was incubated with the corresponding antibody at 4°C overnight with gentle agitation to form the immunocomplex, followed by protein A/G agarose bead incubation under gentle agitation for 1 h at room temperature. Then agarose beads were collected and washed with cell lysis buffer (without protease inhibitor) and eluted with 1× SDS loading buffer. After that, samples were prepared by boiling for 10 min at 98°C and subjected to SDS-PAGE and immunoblotting.

### Pulldown assays.

CIK cells were lysed with cell lysis buffer for Western and IP (Beyotime) supplemented with PMSF (1 mM). Then whole-cell lysates were centrifuged at 12,000 rpm for 20 min at 4°C, the precipitate was discarded, and the supernatant was collected and precleared with Ni-NAT. Precleared cell lysates were incubated with the chelates of His-VP7 protein and Ni-NAT. After that, incubation was carried out at 4°C for 6 h with gentle agitation, followed by bead collection, 3 washings with cell lysis buffer, and resolution by SDS-PAGE-based immunoblotting or mass spectrometry (MS) analysis.

### Transcriptomic analysis.

Transcriptomic sequencing was performed by Novogene Co., Ltd. Briefly, CIK cells or grass carp tissues were treated by RNA isolater (Vazyme) to collect total RNA. The RNA integrity was assessed using the RNA Nano 6000 assay kit of the Bioanalyzer 2100 system (Agilent Technologies, CA, USA) for library preparation and transcriptome sequencing. After that, the clustering of the index-coded samples was performed on a cBot cluster generation system using TruSeq PE cluster kit v3-cBot-HS (Illumina) according to the manufacturer’s instructions. After cluster generation, the library preparations were sequenced on an Illumina NovaSeq platform and 150-bp paired-end reads were generated. The raw data (raw reads) in the fastq format were subjected to quality control, read mapping to the reference genome (Hisat2 v2.0.5), novel transcript prediction (StringTie v1.3.3.b), and quantification of gene level (feature Counts v1.5.0-p3). Then differential expression analysis of two conditions/groups (three biological replicates per condition) was performed using the DESeq2R package (1.20.0), and the Gene Ontology (GO) enrichment analysis of differentially expressed genes was implemented by the cluster Profiler R package, in which gene length bias was corrected; a *P* value less than 0.05 was considered significant enrichment of differentially expressed genes. The heat map and volcano map were drawn by TBtools software with the data obtained according to the above processing method.

### Protein expression and purification.

pCold-TF-EGFP-VP7 plasmids were transformed into the prokaryotic expression system Escherichia coli BL21. Then the expression and purification of the VP7-His tag were performed with the His tag protein purification kit (Beyotime) following the manufacturer’s protocol. Briefly, VP7-His tag protein was induced by 100 μM isopropyl-β-d-thiogalactopyranoside (IPTG) at 16°C overnight in E. coli BL21. After that, the induced E. coli BL21 was collected, subjected to nondenaturing lysis, sonicated with nondenaturing lysis buffer, and then centrifuged at 4°C and 12,000 rpm for 20 min. The supernatant was incubated with BeyoGold His tag purification resin (Beyotime) for 1 h at 4°C with gentle rotation. The bottom cover was removed and the beads were washed 5 times with nondenaturing wash buffer. After that, 5 to 8 washings were done with nondenaturing elution buffer and the effluent was collected for immunoblotting, pulldown, and MS analyses.

### MS analysis.

Mass spectrometry was performed by Sangon Biotech Co., Ltd. Briefly, a reaction mixture containing Tris(2-carboxyethyl)phosphine hydrochloride (TCEP), SDC, and CAA were added to VP7-His beads for alkylation, followed by one-step reduction and elution. These steps were repeated 2 times. Then the eluent was collected and subjected to enzymatic hydrolysis by trypsin. The peptide solution was desalted through a desalting column and drained by a centrifuge concentrator. After that, the samples were analyzed by the Q Exactive Plus liquid mass spectrometry system (Thermo). Mass spectrometry data generated by Q Exactive Plus were retrieved by ProteinPilot (V4.5) using the Paragon database retrieval algorithm.

### Immunofluorescence microscopy and live-cell imaging.

pSDred-N1-VP7 and pEGFP-N1-HSP70 plasmids were transfected into CIK or 293T cells using Invigentech INVI DNA RNA, and cells expressing green or red (GFP or RFP) fluorescent fusion proteins were cultured for 36 h. After that, the cells were fixed with 4% paraformaldehyde for 10 min, stained with 4′,6-diamidino-2-phenylindole (DAPI) for 5 min, washed 3 times with phosphate-buffered saline (PBS), and treated with antifade mounting medium. Then the cells were analyzed with a immunofluorescence microscope (Nikon). For live-cell imaging, GCO cells were seeded on a chambered coverslip at a density of around 50% confluence. Cells were then transfected with corresponding plasmids (pEGFP-N1-HSP70, pDsRed-N1-VP7, pEGFP-N1, and pDsRed-N1) and pictures were recorded per 15 min using a Nikon confocal laser microscope system.

### Electron microscopy.

CIK cells were incubated with or without VP7 for 2 h, and after that, cells were collected for electron microscope observation as previously described [41]. Briefly, CIK cells were immobilized at 4°C by 2.5% glutaraldehyde overnight. After that, the cells were postfixed in 1% osmium tetroxide for 1 h, followed by washing with Dulbecco’s PBS (DPBS), dehydrated, embedded, sectioned, and stained using 2% uranyl acetate and lead citrate. Finally, images were acquired by transmission electron microscopy (H-7650; Hitachi, Tokyo, Japan).

CIK cells were cultured at 28°C overnight and then infected with GCRV; some cells were transferred from 28°C to 18°C for 24 h and the others continued to be incubated at 28°C for 24 h. After that, cells were collected for electron microscopy analysis.

### Statistical analysis.

Statistical analysis was performed using unpaired two-tailed Student’s *t* test or one-way analysis of variance (ANOVA) between different groups. A *P* value of <0.05 was considered statistically significant. In figures, statistical significance is indicated as follows: *, *P < *0.05; **, *P < *0.01; and ***, *P < *0.001. Data are representative of three independent experiments and are shown as the mean ± standard deviations (SD). For heat map analysis and volcano plot, a transcriptomic data matrix normalized by autoscaling was exported into a txt file and analyzed using TBtools software interface with an in-house R script.

### Data availability.

The data that support the findings of this study are available on request from the corresponding author.
