# Supplementary material for: An Aquareovirus Exploits Membrane-Anchored HSP70 To Promote Viral Entry
Source: Microbiol Spectr. 2023 May 9;11(3):e04055-22. doi: 10.1128/spectrum.04055-22 (PMC10269764; doi:10.1128/spectrum.04055-22)
Supplement: Supplemental file 1 — Supplemental material. Download SPECTRUM04055-22_Supp_1_seq11.pdf, PDF file, 0.7 MB [file spectrum.04055-22-s0001.pdf]

Supplementary Materials for

An aquareovirus exploits the membrane anchored HSP70 to promote viral entry

Authors: Guoli Hou, Qiushi Zhang, Chun Li, Geye Ding, Lingling Hu, Xiaoying Chen, Zhao Lv, Yuding Fan, Jun Zou, Tiaoyi Xiao*, Yong-An Zhang*, Junhua Li*

*To whom correspondence should be addressed: yonganzhang@mail.hzau.edu.cn; tiaoyixiao@hunau.edu.cn; lijunhua@hunau.edu.cn

This PDF file includes:

Table S1

Table S2

Figure Legends S1-S4

Supporting videos legends 1-2

Figure S1 to S4

Table S1

REAGENT or RESOURCE	SOURCE	IDENTIFIER
Antibodies		
Rabbit Monoclonal hsp70 antibody	Beyotime	Cat#: AF1156
Rabbit Monoclonal b-actin antibody	Abclonal	Cat#: AC026
Rabbit anti-tubulin mAb	Beyotime	Cat#: AF1216
Rabbit polyclonal integrin antibody	This study	N/A
Mouse polyclonal VP7 antibody	Lab of J Zou	N/A
HRP Goat anti-rabbit antibody	Abclonal	Cat#: AS014
Virus Strains		
GCRV	This study	N/A
Chemicals and Recombinant Proteins		
High-Affinity Ni-NTA Resin	GenScript	Cat#: L00250-25

protein A Sepharose 4 Fast Flow	GE Healthcare	Cat#:17-5280-01
Invigentech INVI DNA RNA	Invigentech	Cat#:40815ES03
Glutaraldehyde 25% aqueous solution	SINOPHARM	Cat#:111-30-8
Recombinant VP7 protein	This study	N/A
Critical Commercial Reagents		
Total RNA Extraction Reagent	Vazyme	Cat#: R401-01
First Strand cDNA Synthesis Kit	Thermo Scientific	Cat#: K1622
SYBR qPCR Master Mix	Vazyme	Cat#: Q711-02
Mem-PER^{TM} Plus Kit	Thermo Scientific	Cat#: VC301678
His-tag Protein Purification Kit	Beyotime	Cat#: P2226
PMSF	Beyotime	Cat#ST505
Imidazole	Solarbio	Cat#I8090
Experimental Models: Cell Lines		
CIK	This study	N/A
GCO	This study	N/A
293T	This study	N/A
Recombinant DNA		
pSDred-N1-VP7	This study	N/A
pSDred-N1-VP7-ΔC	This study	N/A
pSDred-N1-VP7-ΔN	This study	N/A
pEGFP-N1-Hsp70	This study	N/A
pCold-TF-GFP-VP7	This study	N/A
Inhibitor or Activator		
Quercetin (QUE)	AbMole	Cat#:M3902
VER-155008 (VER)	AbMole	Cat#: M3116
LDV peptide	Sangon Biotech	N/A

Table S2

q-PCR primer	Sequence

gc-Hsp70-qF	AAAGACGCCGGAGTAATCGC
gc-Hsp70-qR	TGGTCAGGATGGACACGTCA
gc-βactin-qF	AGCCATCCTTCTTGGGTATG
gc-βactin-qR	GGTGGGGCGATGATCTTGAT
gc-tubulin-qF	ACGGAAGGTGCTGAGCTTGT
gc-tubulin-qR	GACAGCGTGGCGTTGTAAGG
gc-integrin αv-qF	AGCGCATAATGTTCTGTGCTTCA
gc-integrin αv-qR	CCAACCTCCAAGTCACCAAAGTG
gc-lamR-qF	CGGGATCCATGTCCGGAGGTCTGGAT
gc-lamR-qR	CCGCTCGAGAGACCAGTCGGCAGCGGT
gc-SRB1-qF	AGTTTCAGGCATCACAGAAACAGGA
gc-SRB1-qR	GCTGAGGTATAATATCACGGCTCCC
gcMyosin-qF	CCATGGTGGAGCGCAGAAAC
gcMyosin-qR	ATGCACGAGGTCAGCATCCA
gcNF-κb-qF	AGGACACCTTCAGCGACGAG
gcNF-κb-qR	TCTGGATGAGGCTGGCGATG
gc-GCRVII-qF	GTACAGCATTTGGCACGTCT
gc-GCRVII-qR	TCCGCTGAATCGACATACCAC
VP7-qF	CCATGACACTCACGCACACG
VP7-qR	GGCAAGCGAAGGTCAGGTTG

FIG S1 Transcriptomic identification of HSP70 as a key player in GCRV infection

(**A**) Heat map analysis of transcriptomic data (NCBI accession number PRJNA759556) for HSP genes in the liver and kidney tissues from GCRV-infected grass carp. (**B**) Volcano plot analysis of differentially regulated genes from transcriptomic data (NCBI accession number PRJNA600033) in spleen tissue from grass carp infected with GCRV on the 1st, 3rd 7th day. (**C**) Heat map analysis of transcriptomic data for the HSP genes from Fig S1B.

FIG S2 HSP70 mediates GCRV temperature-dependent pathogenesis

(**A**) The relative viral genome replication in the intestine tissue on the 5th day from intraperitoneally GCRV-infected grass carp at 18 °C and 28 °C were analyzed by RT-PCR. (**B**) CIK cells cultured at 18 °C or 28 °C were infected with GCRV (MOI≈5) for 12 h. After then, the cells were harvested with Trizol to analyze the relative viral genome replication and transcription of antiviral genes. (**C-D**) CIK cells cultured at 18 °C, 25 °C , or 28 °C were infected with GCRV (MOI≈5). After then, the cells were harvested at different time points to analyze the relative viral genome replication and transcription of HSP70. (**E**) Schematic of an experimental design depicting that temperature stress regulates HSP70 expression to mediate GCRV infection. CIK cells were cultured at 18 °C or 28 °C for 48 h of adaptation. The cells were then transferred from 18 °C to 28 °C, or from 28 °C to 18 °C, respectively. After that, the cells were infected with GCRV for different time points to quantify the relative viral genome and HSP70 transcription. (**F-G**) CIK cells were prepared for temperature-switch experiments as Fig S2E. The relative GCRV genome replication (**F**) and HSP70 transcription (**G**) at 6 h and 12 h were analyzed by RT-PCR

FIG S3

(**A**) CIK cells cultured at 28°C were treated with different doses of quercetin (0 μM, 1 μM, 10 μM), VER (0 μM, 1 μM, 2 μM) for 2 h. The cell viability was measured with trypan blue assay. (**B-C**) CIK cells were prepared for temperature-switch experiments as Fig S2E. The relative GCRV genome entry and HSP70 transcription at 1 h were analyzed by RT-PCR

FIG S4

(**A**) CIK cells cultured at 28 °C were transfected with different doses of VP7 truncated plasmids (VP7-ΔC deleted C-terminus 91aa, VP7-ΔN deleted N-terminus 80aa,) for 36 h. The cells were then infected with GCRV (MOI≈5) for 1 h to quantify the relative viral genome entry level by RT-PCR. (**B**) CIK cells cultured at 28 °C were transfected with VP7 and/ or VP5 plasmid for 36 h, The cells were then infected with GCRV (MOI ≈5) for 1 h to quantify the relative viral genome entry-level and HSP70 transcription

by RT-PCR. (**C**) The induction of HSP70 transcription by different doses of VP7 plasmids transfection was examined by RT-PCR.

Supporting videos 1-2

CIK cells were co-transfected with pSDred-N1-VP7 and pEGFP-N1-Hsp70 plasmids, Live-cell imaging was recorded per 15 min using Nikon confocal laser microscope system. The red and green signals denote the VP7 (**1**) and HSP70 (**2**) localization, respectively.

Fig S1

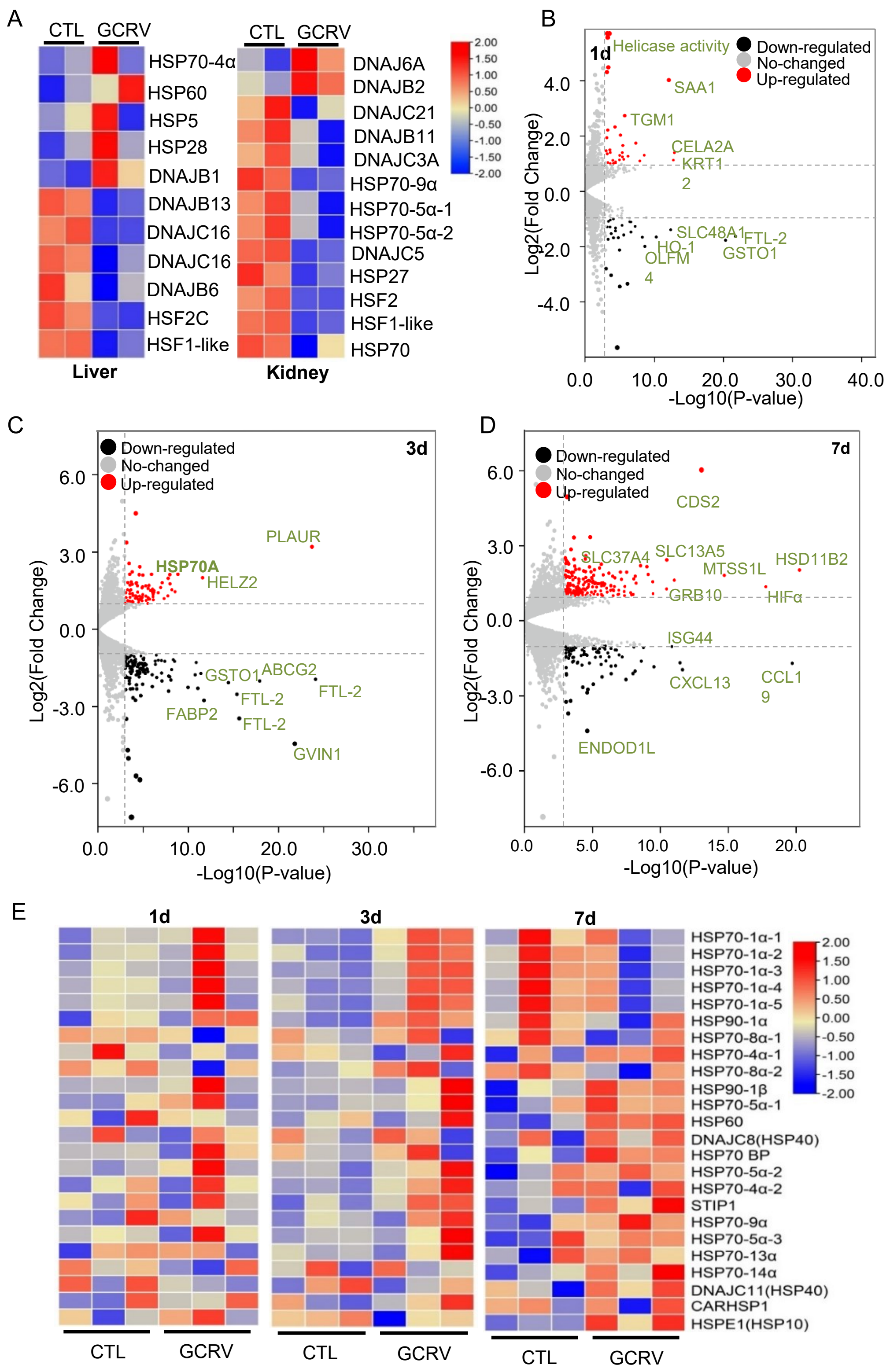
A
CTL GCRV
HSP70-4α
HSP60
HSP5
HSP28
DNAJB1
DNAJB13
DNAJC16
DNAJC16
DNAJB6
HSF2C
HSF1-like
Liver
CTL GCRV
DNAJ6A
DNAJB2
DNAJC21
DNAJB11
DNAJC3A
HSP70-9α
HSP70-5α-1
HSP70-5α-2
DNAJC5
HSP27
HSF2
HSF1-like
HSP70
Kidney
B
1d
Helicase activity
Down-regulated
No-changed
Up-regulated
SAA1
TGM1
CELA2A
KRT1
2
SLC48A1
FTL-2
HO-1
OLFM
4
GSTO1
Log2(Fold Change)
-Log10(P-value)
C
3d
PLAUR
HSP70A
HELZ2
GSTO1
ABCG2
FTL-2
FABP2
GVIN1
D
7d
CDS2
SLC37A4
SLC13A5
MTSS1L
HSD11B2
GRB10
HIFα
ISG44
CXCL13
CCL1
9
ENDOD1L
E
1d
3d
7d
HSP70-1α-1
HSP70-1α-2
HSP70-1α-3
HSP70-1α-4
HSP70-1α-5
HSP90-1α
HSP70-8α-1
HSP70-4α-1
HSP70-8α-2
HSP90-1β
HSP70-5α-1
HSP60
DNAJC8(HSP40)
HSP70 BP
HSP70-5α-2
HSP70-4α-2
STIP1
HSP70-9α
HSP70-5α-3
HSP70-13α
HSP70-14α
DNAJC11(HSP40)
CARHSP1
HSPE1(HSP10)
CTL GCRV

Fig S2

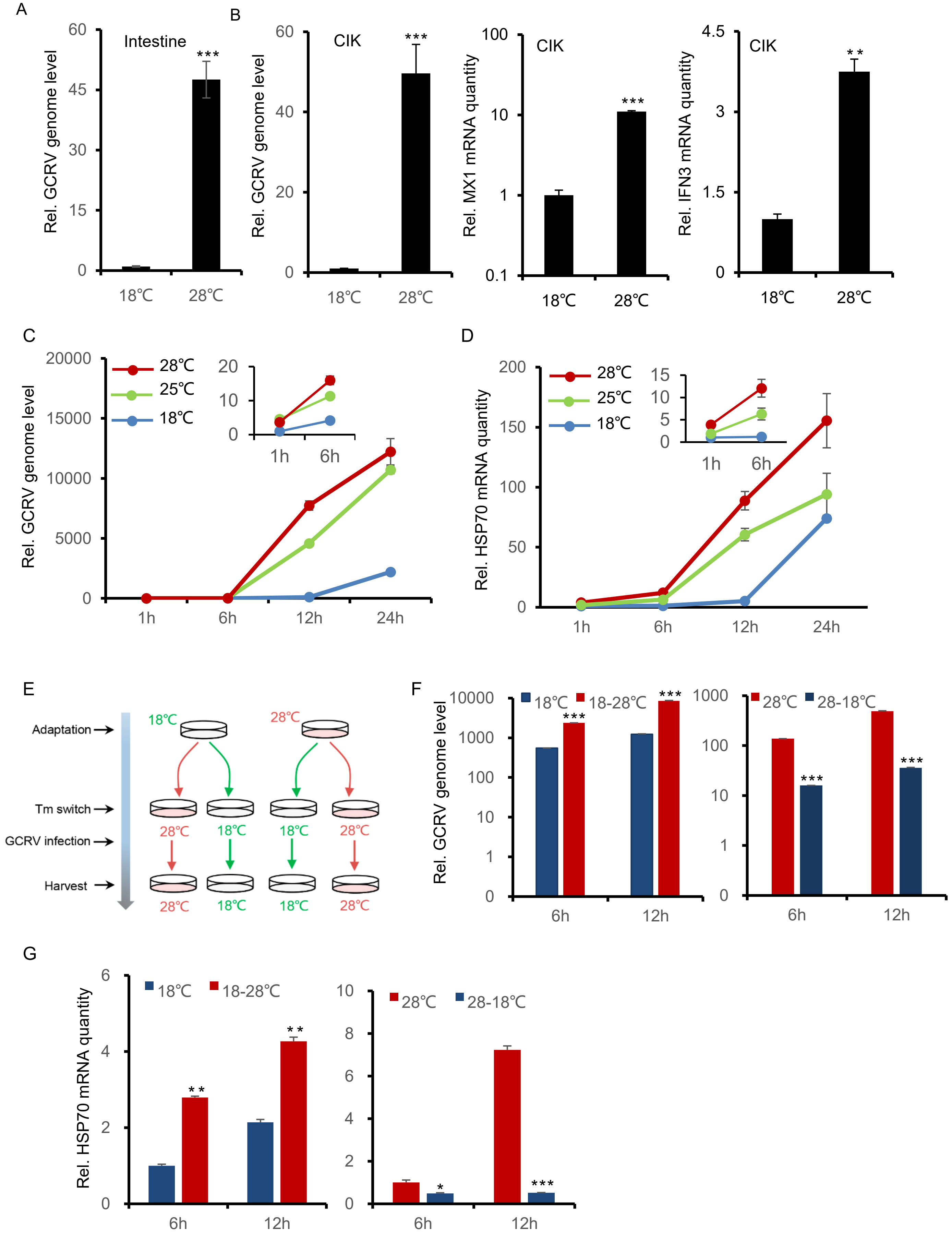
A
Intestine
Rel. GCRV genome level
18℃
28℃
***
B
CIK
Rel. GCRV genome level
Rel. MX1 mRNA quantity
Rel. IFN3 mRNA quantity
C
Rel. GCRV genome level
28℃
25℃
18℃
1h
6h
12h
24h
D
Rel. HSP70 mRNA quantity
E
Adaptation
Tm switch
GCRV infection
Harvest
F
18℃
18-28℃
28℃
28-18℃
Rel. GCRV genome level
G
Rel. HSP70 mRNA quantity

Fig S3

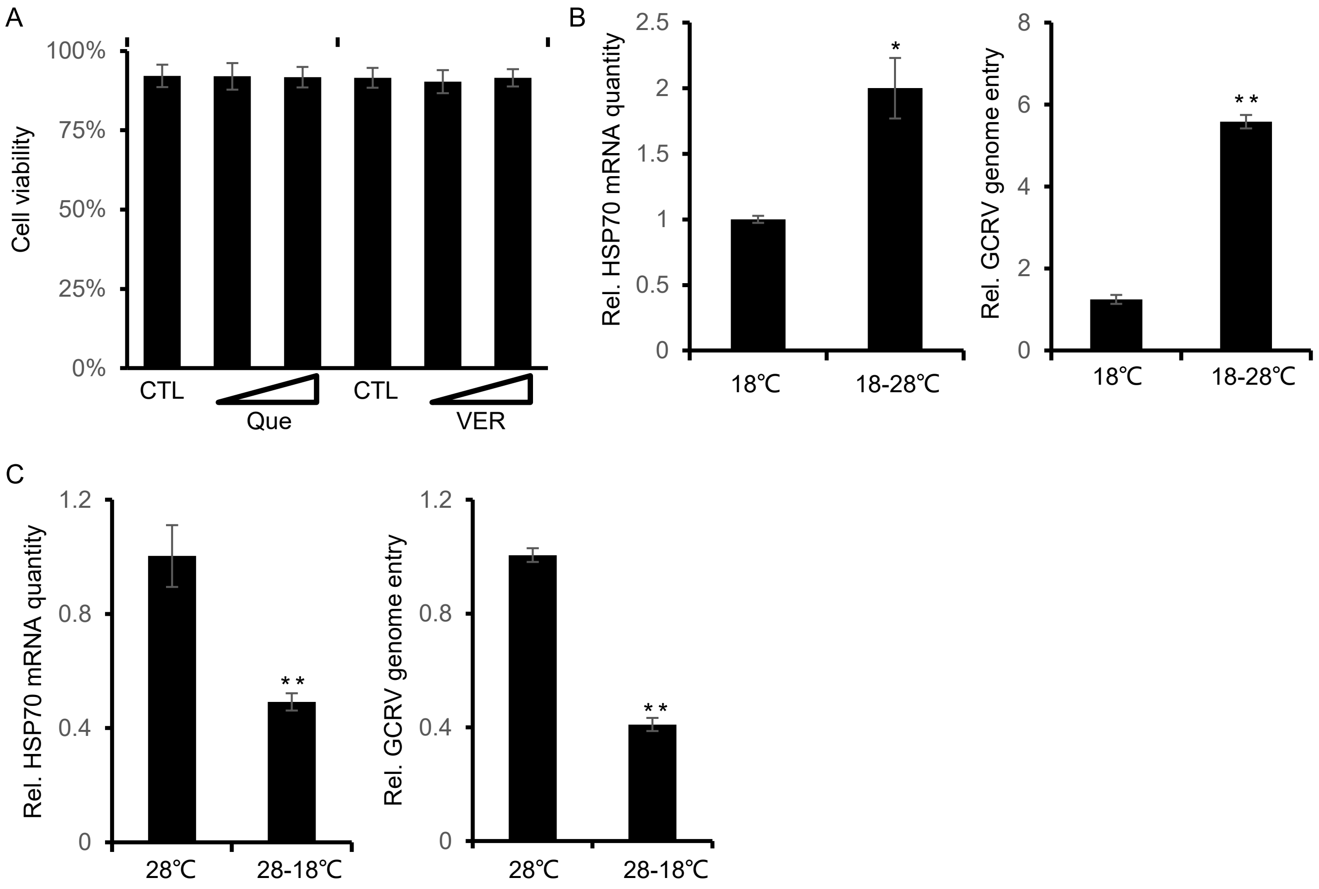

A
Cell viability
100%
75%
50%
25%
0%
CTL
Que
CTL
VER
B
Rel. HSP70 mRNA quantity
2.5
2
1.5
1
0.5
0
*
18℃
18-28℃
Rel. GCRV genome entry
8
6
4
2
0
* *
18℃
18-28℃
C
Rel. HSP70 mRNA quantity
1.2
0.8
0.4
0
* *
28℃
28-18℃
Rel. GCRV genome entry
1.2
0.8
0.4
0
* *
28℃
28-18℃

Fig S4

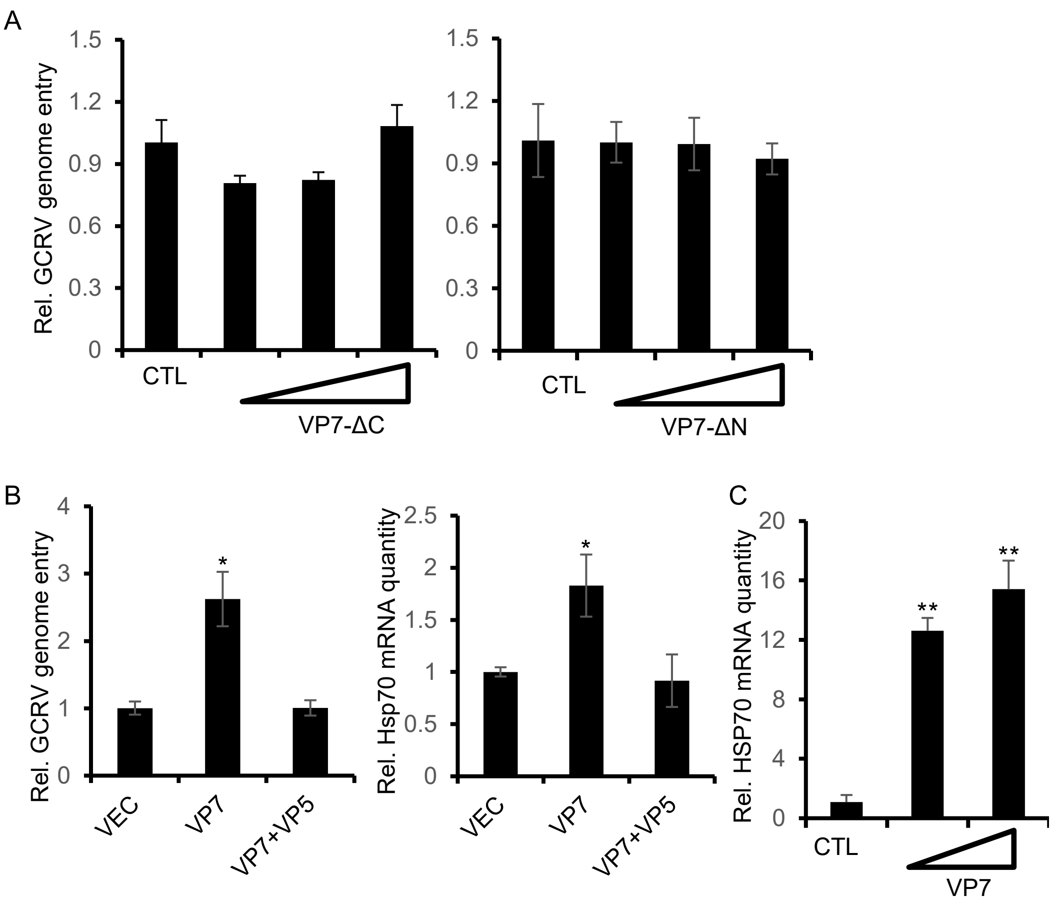
A
Rel. GCRV genome entry
1.5
1.2
0.9
0.6
0.3
0
CTL
VP7-ΔC
1.5
1.2
0.9
0.6
0.3
0
CTL
VP7-ΔN
B
Rel. GCRV genome entry
4
3
2
1
0
*
VEC
VP7
VP7+VP5
Rel. Hsp70 mRNA quantity
2.5
2
1.5
1
0.5
0
*
VEC
VP7
VP7+VP5
C
Rel. HSP70 mRNA quantity
20
16
12
8
4
0
**
**
CTL
VP7
